# *HLA-A* Confers an *HLA-DRB1* Independent Influence on the Risk of Multiple Sclerosis

**DOI:** 10.1371/journal.pone.0000664

**Published:** 2007-07-25

**Authors:** Boel Brynedal, Kristina Duvefelt, Gudrun Jonasdottir, Izaura M. Roos, Eva Åkesson, Juni Palmgren, Jan Hillert

**Affiliations:** 1 Division of Neurology, Department of Clinical Neuroscience, Karolinska Institutet, Stockholm, Sweden; 2 Mutation Analysis Facility, Clinical Research Center, Karolinska University Hospital, Stockholm, Sweden; 3 Department of Medical Epidemiology and Biostatistics, Karolinska Institutet, Stockholm, Sweden; Baylor College of Medicine, United States of America

## Abstract

A recent high-density linkage screen confirmed that the HLA complex contains the strongest genetic factor for the risk of multiple sclerosis (MS). In parallel, a linkage disequilibrium analysis using 650 single nucleotide polymorphisms (SNP) markers of the HLA complex mapped the entire genetic effect to the *HLA-DR-DQ* subregion, reflected by the well-established risk haplotype HLA-DRB1*15,DQB1*06. Contrary to this, in a cohort of 1,084 MS patients and 1,347 controls, we show that the *HLA-A* gene confers an *HLA-DRB1* independent influence on the risk of MS (P = 8.4×10^−10^). This supports the opposing view, that genes in the HLA class I region indeed exert an additional influence on the risk of MS, and confirms that the class I allele HLA-A*02 is negatively associated with the risk of MS (OR = 0.63, P = 7×10^−12^) not explained by linkage disequilibrium with class II. The combination of *HLA-A* and *HLA-DRB1* alleles, as represented by HLA-A*02 and HLA-DRB1*15, was found to influence the risk of MS 23-fold. These findings imply complex autoimmune mechanisms involving both the regulatory and the effector arms of the immune system in the triggering of MS.

## Introduction

The human leukocyte antigen (HLA) gene complex on chromosome 6 has been linked to and associated with several supposedly autoimmune diseases. This highly variable region harbors over 420 genes[1] and has been unambiguously associated with multiple sclerosis (MS, MIM 126200) susceptibility since 1972[2]. Extensive polymorphism and linkage disequilibrium (LD) complicates the efforts to precisely map associations with MS in this region but most evidence has pointed to the class II region and the *HLA-DRB1*(GeneID: 3123) and *-DQB1* genes, and specifically to the haplotype DRB1*1501,DRB5*0101,DQA1*0102,DQB1*0602 [3].

Recently, Lincoln and colleagues reported data on 4,203 individuals from 1,185 Canadian and Finnish MS families genotyped for approximately 650 SNP markers spanning the HLA complex and its flanking regions, in all 13 Mb[4]. Strong associations were observed with several LD blocks within the HLA class II subregion. Conditioning for *HLA-DRB1* revealed that all block or SNP associations were dependent on the *HLA-DRB1* gene, implying that the HLA associated MS susceptibility is determined by HLA class II alleles or closely located variants [4]. However, this study did not assess any functional gene variants besides *HLA-DRB1*, such as the *HLA-A* gene (GeneID: 3105). Also, most likely due to the difficulty of designing assays against SNP markers in highly polymorphic genes, no SNP within or close to *HLA-A* were genotyped. The original reports on HLA associations with MS were indeed focused on the HLA class I specificities *HLA-A* and *HLA-B* [2], [5]. Somewhat later, when class II specificities were discovered and the strong association between *HLA-DRB1* and MS emerged, the class I associations were regarded as secondary [6]. More recently the possible importance of HLA class I genes in MS susceptibility has been re-investigated by us and others [7]–[9]. Fogdell-Hahn and co-workers examined the role of classical HLA genes including both class I (*HLA-A, -B, -C*) and class II (*HLA-DRB1, -DQ, -DP*), indicating that HLA-A*03 confers a risk for MS while HLA-A*02 has a protective effect [7]. This primary study, as well as that of Harbo and co-workers [8], was too limited in size to establish a possible independence from the influence of class II alleles due to LD. Further, in addition to the classical association with the HLA-DRB1*15-carrying haplotype, recent studies have identified a more complex situation where several *HLA-DRB1* alleles interact in determining the risk of MS [10], [11]. In the present study, we set out to investigate how alleles of the *HLA-DRB1* and *HLA-A* genes interact in determining the risk of MS. In order to distinguish the importance of two multiallelic loci, in addition to a sufficiently large sample size, a statistic strategy needs to be applied where the effect of one allele can be evaluated in the presence of other effects. Here, we use multiple logistic regression models enabling simultaneous analysis of all included alleles while adjusting for the potential confounding effect due to LD [12]. Thus, we were able to establish an *HLA-DRB1* independent effect of *HLA-A* in MS susceptibility.

## Materials and Methods

The 1,084 MS patients included in this study were diagnosed by neurologists at the Karolinska University Hospital and fulfilled the McDonald criteria of MS[13], their mean age was 51.5 years. The control group consisted of 1,347 consecutively collected blood donors with a mean age of 45.6. The study population was residents of the Stockholm area and predominantly Swedish, including a few percent from other Scandinavian countries but excluding persons with a non-Scandinavian origin. Oral informed consent was obtained from all participants, and the ethical board of Karolinska Institutet approved the study. None of the included patients or controls had been included in our previous study on HLA class I in MS [7].

Functional alleles of *HLA-A* and *HLA-DRB1* were identified by use of allele specific primers [14] (Table 1). Genotypes carried by individuals utilized as controls were in Hardy Weinberg equilibrium [15] for both genes (data not shown). For the logistic regression analysis, we used the methodology proposed by Cordell and Clayton [12] in which nested hypotheses involving logistic regression models of different loci are compared using likelihood ratio tests. The first step was performed to establish which locus had the strongest effect in initial models with single point effects of *HLA-A* and *HLA-DRB1*. The following step involved adding loci into the model until the Likelihood-Ratio Test (LRT) was non-significant. In our analysis this meant assessing the additional effect of *HLA-A* on MS susceptibility over and above the effect of *HLA-DRB1*. The analysis up to this point included all alleles with a frequency among the MS patients of >5%.

**Table 1:** Allele frequencies (at a resolution corresponding to serological specificities) of *HLA-A* and *HLA-DRB1* in MS patients (n = 1084) and healthy controls (n = 1347).

	MS	HC
HLA-	frequency (%)	frequency (%)
A*01	14.4	13.6
A*02	26.6	35.9
A*03	21.2	17.5
A*11	5.7	5.1
A*24	9.8	8.2
***AX*** a	22.4	19.8
DRB1*01	6.8	11.3
DRB1*03	11.5	12.5
DRB1*04	16.0	18.9
DRB1*08	5.1	5.4
DRB1*13	10.9	14.3
DRB1*15	35.5	15.6
***DRB1X*** b	22.2	14.4

a AX includes all observed alleles at the *HLA-A* locus with frequencies of less than 5% in cases; A*23, A*25, A*26, A*29, A*30, A*31, A*32, A*33, A*66, A*68, A*69, A*74 and A*210.

b DRB1X includes all observed alleles at the *HLA-DRB1* locus with frequencies of less than 5% in cases;DRB1*07, DRB1*09, DRB1*10, DRB1*11, DRB1*12, DRB1*14, DRB1*16 and DRB1*103.

To further pinpoint the specific alleles contributing to MS susceptibility, we applied stepwise logistic regression on an allelic level. Starting from a model with all *HLA-A* and *HLA-DRB1* alleles, the least significant allele was removed one at the time, until the effect size of all remaining alleles were significant. Finally, the last model comparison assessed whether an interaction between *HLA-DRB1* and *HLA-A* would improve the prediction. For a complete description of all the models used in the analysis see Supporting Information Table S1. It should be noted that odds ratios obtained in a logistic regression framework are adjusted for all the other alleles included in the model, and therefore differ from those obtained when a given allele is compared to all other alleles, the most commonly used comparison in previous studies. We declared the odds ratio for a specific allele (relative to the baseline comparators) as statistically significant if the p-value exceeded a Bonferroni corrected threshold. The overall significance level α, chosen to be 0.05, was divided by 11, i.e. the total number of global tests (4) plus the total number of steps in the stepwise allele selection (7).

## Results and Discussion

A model including the main effects of both *HLA-A* and *HLA-DRB1* (model 3 in Table 2) predicted the subjects' disease status significantly better than the model including only *HLA-DRB1* (model 2). The P-value for this comparison was 8×10^−10^, illustrating a strong additional effect of *HLA-A* on MS susceptibility (Table 2), It should be noted that the P-value refers to the *independent* effect of *HLA-A*, since the LD between the two loci is accounted for by including (conditioning on) *HLA-DRB1* in the logistic regression model.

**Table 2:** Comparison of the nested logistic regression models of HLA-DRB1 and HLA-A

Model	Model terms	Deviance	Df^a^	Model comparison	ΔDeviance^b^	ΔDf^c^	P-value
**0**		3336.4	2426				
**1**	HLA-A^d^	3286.1	2421	Model 1 vs. 0	50.3	5	**1.2×10^−9^**
**2**	*HLA-DRB1* e	3054.1	2420	Model 2 vs. 0	282.3	6	**5.1×10^−58^**
**3**	*HLA-DRB1*+*HLA-A* f	3003.1	2415	Model 3 vs. 2	51.05	5	**8.4×10^−10^**
**4**	HLA-DRB1+HLA-A^g^	3008.05	2422				
**5**	HLA-DRB1 * HLA-A h	3007.87	2419	Model 5 vs. 4	0.19	3	**0.98**

a Df is the degrees of freedom in the model.

b The Δ Deviance is the difference is deviance between the compared models. A large value (small P-value) indicates that the smaller models does not predict the outcome well, thus the smaller model is rejected.

c ΔDf is the difference in degrees of freedom between the compared models, i.e. the number of parameters restricted in the smaller model.

d Includes main effects of all alleles at *HLA-A*.

e Includes main effects of all alleles at *HLA-DRB1*,

f Includes main effects of all alleles at *HLA-A* and *-DRB1*.

g Reduced model with main effects of significantly associated alleles at *HLA-A* and *-DRB1*

h Includes main effects, as well as interaction effects of significantly associated alleles at *HLA-A* and *-DRB1*

In order to pinpoint the alleles that confer an effect on MS susceptibility, a stepwise logistic regression was performed on all the alleles with frequency above 5%. The excluded alleles are reported in Supporting Information Table S2, whereas the alleles in the final model are reported in Table 3 along with corresponding statistics. As expected, our results showed that the DRB1*15 allele increased the risk of developing MS (OR = 2.9, P<2×10^−16^) (Table 3). The described heterogeneity of effects on MS susceptibility at the *HLA-DRB1* locus [10], [11] was partly confirmed, since several DRB1 alleles show significant association (Table 3). Dyment et al [10] reported an under-transmission of DRB1*01 to affected offspring positive for DRB1*15, thus the DRB1*01/DRB1*15 genotype would be uncommon among patients with MS. In contrast, we observed a protective effect of HLA-DRB1*01 (OR = 0.69 P = 1×10^−3^), independently of HLA-DRB1*15. Similarly, a pool of less common *HLA-DRB1* alleles (DRB1*07, DRB1*09, DRB1*10, DRB1*11, DRB1*12, DRB1*14, DRB1*16 and DRB1*103) was negatively associated with the risk of MS (OR = 0.77, P = 2×10^−3^). This was most likely an effect of one or several of the included alleles. All DRB1X alleles had low frequency and were combined into one entity due to expected lack of power.

**Table 3:** Alleles of *HLA-A* and *HLA-DRB1* significantly associated with the susceptibility to MS identified by backward stepwise logistic regression analysis.

Allele	OR	P-value
HLA-A*02	0.63	6.8×10^−12^
HLA-DRB1*01	0.69	1.2×10^−3^
HLA-DRB1*15	2.9	<2×10^−16^
*HLA-DRB1X*	0.77	1.6×10^−3^

At the *HLA-A* locus, the A*02 allele decreased the risk of MS (OR = 0.63, P = 7×10^−12^). No other *HLA-A* allele had an effect that differed significantly from that of the baseline.

Our results do not confirm an independent role of HLA-A*03 in MS susceptibility. In Table 4 we have compared our results, using logistic regression, with Fishers exact test and Cochran-Armitage test for trend of genotype distribution in our material. Both unadjusted approaches showed significant p-values for HLA-A*03 (3×10^−3^ and 7×10^−4^ respectively), whereas adjusting for other alleles generated a p-value of 0.95. This may seem contradictory to previous studies [7], [8], but in fact shows the strength of the current statistical approach; the A*03 association previously detected is presumably a reciprocal effect of the decreased A*02 frequency or caused by confounding from the *DRB1* locus. At the *HLA-DRB1* locus, DRB1*03, DRB1*04 and DRB1*13 show significant p-values in the unadjusted analysis, not confirmed by logistic regression, thus most likely a reciprocal effect of the strong DRB1*15 association.

**Table 4:** Comparison of p-values: logistic regression with all alleles of *HLA-DRB1* and *HLA-A* included simultaneously vs. Cochran-Armitage Trend tests and Fisher's Exact tests on one allele at the time.

HLA allele	P-value^a^	Armitage^b^	Fisher's^c^
A*01		0.43	0.06
A*02	6.8×10^−12^	9.6×10^−12^	4.4×10^−11^
A*03	0.95^d^	6.9×10^−4^	2.7×10^−3^
A*11	0.52^d^	0.34	0.47
A*24	0.48^d^	0.04	0.11
***AX***	0.38^d^	0.03	0.07
DRB1*01	1.3×10^−3^	1.4×10^−7^	6.3×10^−7^
DRB1*03	0.41^d^	0.27	1.8×10^−4^
DRB1*04	0.14^d^	8.4×10^−3^	1.9×10^−3^
DRB1*08		0.72	0.81
DRB1*13	0.46^d^	5.0×10^−4^	1.0×10^−4^
DRB1*15	<2×10^−16^	0	3.3×10^−58^
***DRB1X***	1.6×10^−3^	1.1×10^−11^	3.6×10^−11^

a P-values from the stepwise logistic regression analysis.

b P-value from Cochran-Armitage test of trend of genotype distribution.

c P-value from Fisher's exact test of genotype distribution.

d The exclusion p-values from the stepwise logistic regression, see Table S1.

We proceeded by fitting a model with possible interactions between the *HLA-A* and *HLA-DRB1* loci. When comparing the model including interaction terms (model 5) with model 4 we found no improvement in the ability to predict the subjects' disease status (Table 2); the global p-value was 0.98 and none of the included interaction terms were significant. We could therefore conclude that the HLA-A*02 association was independent of the *HLA-DRB1* locus and specifically the DRB1*15 allele; neither LD nor interaction with *HLA-DRB1* had influenced the association of HLA-A*02 with MS. So in contrary to the recent SNP-based LD mapping study [4], we clearly show a HLA class I effect independent of *HLA-DRB1*.

**Figure 1:**
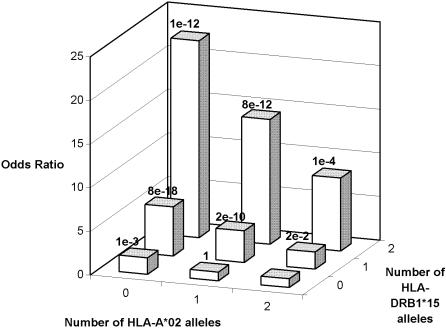
Empirical odds ratios (ORs) for combinations of the HLA-DRB1*15 and HLA-A*02 alleles. A genotype of two HLA-A*02 alleles but no HLA-DRB1*15 allele was used as baseline in the calculation of ORs. P values are reported above the bars.

It has previously been reported that HLA-DRB1*15 exerts an allele dose effect on the risk of MS [16], [17]. Figure 1 illustrates how the genotypes of *HLA-DRB1* and *HLA-A* jointly modified the empirical OR's of MS, the most susceptible genotypes (homozygosity for DRB1*15 but no A*02) and most resistant (homozygosity for A*02 but no DRB1*15) genotypes differed 23 fold in the risk of MS. The magnitude of the risk jointly conferred by these loci greatly exceeds the relative risk of 3.5 typically seen for carriage of HLA-DRB1*15. This indicates that genes in the HLA region may confer a larger fraction of the genetic aetiology of MS than previously thought. This notion is further supported by the results of recent MS linkage analyses; in these studies, with increasing power of the analysis, the HLA locus has gradually obtained higher LOD scores, eventually exceeding 11, while all other candidate loci have remained insignificant[18], [19]. Recently, Yeo and co-workers reported an association with several alleles of *HLA-DRB1,* together with a negative association with the HLA class I allele HLA-C*05 in patients lacking DRB1 risk alleles, a finding which supports the importance of HLA class I genes in MS [9]. However, it remains to be studied whether the *HLA-C* locus contributes to MS susceptibility in our population, and, if so, whether there may be a confounding effect of LD between HLA-A*02 and HLA-C*05.

The HLA complex is reputed for strong LD. Therefore, a newly reported genetic association could be expected to be secondary to a previously known association. As explained above, this was clearly not the case for the *HLA-A* association. The two global measures used (Cramer's V and Kendall's tau-b) showed low levels of LD (0.25 and -0.030 respectively). However weak, an LD of this magnitude could potentially affect allele distributions at the linked locus and induce a secondary association, but in the case of the HLA-A*02 allele this possibility is rejected by the results of the logistic regression analysis (P = 8.4×10^−10^). Pairwise LD has been estimated and is reported in Supporting Information Tables S3 and S4. While it is principally possible that the *HLA-A* association in MS is due to another genetic variant in LD with HLA-A*02, data from the HapMap project shows that *HLA-A* is not part of any sizable LD block.

Frequencies of HLA alleles are known to vary considerably between different populations, presumably due to genetic drift and different environmental challenges. Therefore a correct random sample of the population is important for the evaluation of HLA associations within that population. We therefore compared the distribution of *HLA-A* and *HLA-DR* alleles in our controls to the HLA genotype data available from the Swedish bone marrow registry (n = 40 162 and 11 006 respectively) and found no significant deviation. In particular, the HLA-A*02 frequencies are highly comparable (allele frequency of 35.9% in our controls compared to 35.3% in the Swedish bone marrow registry). Thus we feel confident that our control group is a suitable random sample of the Swedish population.

The HLA gene complex is different from other genomic regions by harbouring the most polymorphic genes we know; isotypic and allotypic polymorphism is in fact central to the physiological role of the class I and class II molecules. Therefore, it is not surprising that genetic association studies applying anonymous markers such as microsatellites and SNPs [4] are less efficient in detecting associations than the functional variants themselves.

With the present results in mind, it is reasonable to speculate that the genetic risk is indeed influenced by a function of an HLA class I molecule, possibly HLA-A. In contrast to the principle role of HLA-class II molecules in the triggering of an adaptive immune response, HLA class I antigens instead typically interact with cytotoxic CD8+ T cells. In fact, CD8+ T cells outnumber CD4+ T cells in MS lesions and may be of central importance in lesion pathogenesis (reviewed in [20]). In addition, HLA class I molecules also interact with NK cells, and are in this way important for innate immunity.

Further mapping efforts to locate genetic effects on MS susceptibility within this region should preferably employ functional variants along with other markers. In addition, functional studies of HLA class I molecules in MS are motivated.

To conclude; using a large case control material and simple, straightforward statistical analysis we have shown that *HLA-A* confers an additional influence on MS susceptibility in the Swedish population, not attributable to the known *HLA-DRB1* association. This role of HLA class I genes may have important implications for the disease triggering mechanisms in MS.

## Supporting Information

Table S1 Model description.
(0.03 MB DOC)

Table S2 Sequential exclusion of alleles in the stepwise logistic regression procedure. At each step, the least significant allele was removed until all remaining alleles in the model were significant (Table 3) at α  = 0.05/11.
(0.03 MB DOC)

Table S3 Pair-wise LD measures, D' and R2, and two global (multi-allelic) measures of LD: Cramer's V and Kendall's tau-b for alleles of HLA-A and HLA-DRB1 among controls.
(0.05 MB DOC)

Table S4 Pair-wise LD measures, D' and R2, and two global (multi-allelic) measures of LD: Cramer's V and Kendall's tau-b for alleles of HLA-A and HLA-DRB1 among cases.
(0.05 MB DOC)
