# Supplementary material for: HLA-A Confers an HLA-DRB1 Independent Influence on the Risk of Multiple Sclerosis
Source: PLoS One. 2007 Jul 25;2(7):e664. doi: 10.1371/journal.pone.0000664 (PMC1919434; doi:10.1371/journal.pone.0000664)
Supplement: Table S1 — Model description. (0.03 MB DOC) [file pone.0000664.s001.doc]

**Supporting Information Table S1**. Model description.

| **Model no** | **Model description** |
| --- | --- |
| Model 0 | logit(p) = β_0_ |
| Model 1 | logit(p) = β_0_ + β_1_ A*02 + β_2_ A*03 + β_3_ A*11 + β_4_ A*24 + β_5_ AX = β_0_ + **β**_1A_ **A** |
| Model 2 | logit(p) = β_0_ + β_6_ DRB1*01 + β_7_ DRB1*03 + β_8_ DRB1*04 + β_9_ DRB1*13 + β_10_ DRB1*15 + β_11_ DRB1X = β_0_ + **β**_1DR_ **DRB1** |
| Model 3 | logit(p) = β_0_ + **β**_1A_ **A** + **β**_1DR_ **DRB1** |
| Model 4 | logit(p) = β_0_ + β_1_ A*02 + β_2_ DRB1*01 + β_3_ DRB1*15 + β_4_ DRB1X |
| Model 5 | logit(p) = β_0_ + β_1_ A*02+ β_2_ DRB1*01 + β_3_ DRB1*15 + β_4_ DRB1X + β_5_ A*02xDRB1*01 + β_6_ A*02xDRB1*15 + β_7_ A*02xDRB1X |

p denotes the probability of MS, given the specific alleles at *HLA-A* and *HLA-DRB1*.

A*02, A*03, etc and DRB1*01, DRB1*03, etc refers to the allele count of that particular allele.

The *HLA-A*01* and *-DRB1*08* allele were set as baseline in models 1, 2 and 3, since they had comparable frequencies in cases and controls.
