# Supplementary material for: HLA-A Confers an HLA-DRB1 Independent Influence on the Risk of Multiple Sclerosis
Source: PLoS One. 2007 Jul 25;2(7):e664. doi: 10.1371/journal.pone.0000664 (PMC1919434; doi:10.1371/journal.pone.0000664)
Supplement: Table S2 — Sequential exclusion of alleles in the stepwise logistic regression procedure. At each step, the least significant allele was removed until all remaining alleles in the model were significant (Table 3) at α = 0.05/11. (0.03 MB DOC) [file pone.0000664.s002.doc]

**Supporting Information Table S2.** Sequential exclusion of alleles in the stepwise logistic regression procedure. At each step, the least significant allele was removed until all remaining alleles in the model were significant (Tabel 3) at α = 0.05/11.

| Step | Removed allele | Corresponding p-value |
| --- | --- | --- |
| 1 | *HLA-A*03* | 0.95 |
| 2 | *HLA-A*11* | 0.52 |
| 3 | *HLA-A*24* | 0.48 |
| 4 | *HLA-AX* | 0.38 |
| 5 | *HLA-DRB1*04* | 0.15 |
| 6 | *HLA-DRB1*03* | 0.41 |
| 7 | *HLA-DRB1*13* | 0.46 |
