# Supplementary material for: HLA-A Confers an HLA-DRB1 Independent Influence on the Risk of Multiple Sclerosis
Source: PLoS One. 2007 Jul 25;2(7):e664. doi: 10.1371/journal.pone.0000664 (PMC1919434; doi:10.1371/journal.pone.0000664)
Supplement: Table S4 — Pair-wise LD measures, D' and R2, and two global (multi-allelic) measures of LD: Cramer's V and Kendall's tau-b for alleles of HLA-A and HLA-DRB1 among cases. (0.05 MB DOC) [file pone.0000664.s004.doc]

**Supporting Information Table S4.** Pair-wise LD measures, D’ and R^2^, and two global (multi-allelic) measures of LD: Cramer’s V and Kendall’s tau-b for alleles of *HLA-A* and *HLA-DRB1* among cases.

|  | D-prime | | | | | | |  | R-square | | | | | | |
| --- | --- | --- | --- | --- | --- | --- | --- | --- | --- | --- | --- | --- | --- | --- | --- |
|  | **DRB1*01** | **DRB1*03** | **DRB1*04** | **DRB1*08** | **DRB1*13** | **DRB1*15** | **DRB1X^a^** |  | **DRB1*01** | **DRB1*03** | **DRB1*04** | **DRB1*08** | **DRB1*13** | **DRB1*15** | **DRB1X^a^** |
| **A*01** | 0.69 | 0.40 | 0.46 | 0.64 | 0.50 | 0.30 | 0.73 |  | 5.9 e-3 | 0.20 | 6.7 e-3 | 3.7 e-3 | 5.1 e-3 | 8.0 e-3 | 1.5 e-2 |
| **A*02** | 0.37 | 0.78 | 0.47 | 3.4 e-2 | 2.2 e-2 | 3.3 e-2 | 3.2 e-3 |  | 3.7 e-3 | 2.9 e-2 | 4.1 e-3 | 7.9 e-3 | 1.5 e-3 | 1.7 e-3 | 2.3 e-5 |
| **A*03** | 2.7 e-2 | 0.49 | 0.28 | 4.8 e-2 | 0.11 | 0.12 | 0.31 |  | 2.6 e-3 | 8.4 e-3 | 4.0 e-3 | 3.3 e-5 | 4.0 e-4 | 3.1 e-2 | 4.4 e-3 |
| **A*11** | 1.5 e-3 | 0.41 | 2.7 e-2 | 0.30 | 4.7 e-2 | 1.7 e-2 | 6.9 e-3 |  | 2.9 e-6 | 1.3 e-3 | 2.3 e-3 | 2.9 e-4 | 1.6 e-5 | 9.3 e-6 | 1.3 e-4 |
| **A*24** | 0.60 | 0.35 | 0.10 | 0.19 | 4.4 e-2 | 4.0 e-2 | 5.5 e-2 |  | 2.8 e-3 | 1.7 e-3 | 2.1 e-4 | 2.2 e-4 | 2.2 e-3 | 9.4 e-5 | 4.7 e-3 |
| **AX^b^** | 5.2 e-2 | 0.32 | 3.6 e-2 | 0.24 | 8.0 e-3 | 0.27 | 7.6 e-2 |  | 1.0 e-2 | 3.8 e-3 | 1.9 e-3 | 8.8 e-4 | 1.5 e-4 | 1.1 e-2 | 9.8 e-3 |
| Cramer's V = 0.2311 | | |  |  |  |  |  |  |  |  |  |  |  |  |  |
| Kendall's tau-b = -0.0371 ASE = 0.017 | | | | |  |  |  |  |  |  |  |  |  |  |  |

^a^ DRB1X includes all observed alleles at the *HLA-DRB1*  locus with frequency of less than 5 % in cases; DRB1*07, DRB1*09, DRB1*10, DRB1*11, DRB1*12, DRB1*14, DRB1*16 and DRB1*103

^b^ AX includes all observed alleles at the *HLA-A* locus with frequency of less than 5 % in cases; A*23, A*25, A*26, A*29, A*30, A*31, A*32, A*33, A*66, A*68, A*69, A*74 and A*210.
